# Phenotypic and Genetic Associations Between Cardiovascular Disease Subtypes and Alzheimer’s Disease

**DOI:** 10.1101/2025.08.29.25334750

**Published:** 2025-09-02

**Authors:** Aili Toyli, Chen Zhao, Kuan-Jui Su, Hui Shen, Hong-Wen Deng, Qing-Hui Chen, Qiuying Sha, Weihua Zhou

**Affiliations:** 1. Department of Mathematical Sciences, Michigan Technological University, Houghton, MI, USA 49931; 2. Department of Computer Science, Kennesaw State University, 680 Arntson Dr, Marietta, GA, USA 30060; 3. Division of Biomedical Informatics and Genomics, Tulane Center of Biomedical Informatics and Genomics, Deming Department of Medicine, Tulane University, New Orleans, LA, USA 70112; 4. Department of Kinesiology and Integrative Physiology, Michigan Technological University, Houghton, MI, USA; 5. Department of Applied Computing, Michigan Technological University, 1400 Townsend Dr, Houghton, MI, USA 49931; 6. Center for Biocomputing and Digital Health, Institute of Computing and Cybersystems, and Health Research Institute, Michigan Technological University, Houghton, MI, USA 49931

**Keywords:** Heart–brain axis, Alzheimer’s disease, cardiovascular disease, hypotension, cerebral infarction

## Abstract

**Background:**

Cardiovascular disease (CVD) and Alzheimer’s disease (AD) are major public health concerns that share overlapping risk factors and potential mechanistic pathways. While vascular contributions to cognitive decline are well-documented, the specific relationships between AD and different CVD subtypes remain poorly understood.

**Methods:**

We examined associations between AD and 11 CVD subtypes using logistic regression models in two large biobanks: the UK Biobank (n = 502,133) and the All of Us Research Program (n = 287,011). Models were adjusted for demographic, lifestyle, and clinical covariates. We also explored genetic overlap between AD and CVD traits through colocalization of significant single nucleotide polymorphisms (SNPs) (p < 5×10^−8^) using genome-wide association study (GWAS) data.

**Results:**

Most CVD subtypes were significantly associated with AD in both cohorts. Hypotension had the strongest and most consistent association, followed by hypertension and cerebral infarction. Acute myocardial infarction was the only subtype not significantly linked to AD. Genetic analyses revealed shared loci between AD and CVD-related traits, particularly in regions near *APOE, MAPT*, and genes influencing myocardial structure and vascular function.

**Conclusions:**

This study identifies subtype-specific CVD associations with AD across two diverse cohorts and highlights shared genetic architecture underlying heart–brain interactions. These findings underscore the importance of vascular health in AD risk and suggest that certain CVD subtypes, especially hypotension, may play underrecognized roles in cognitive decline.

## Introduction

A growing body of evidence supports a strong and bidirectional relationship between cardiovascular and brain health^1^. The brain relies heavily on the heart, receiving 15% of the body’s cardiac output and 20% of its oxygen supply^2^, while the heart’s function is regulated by the nervous system^3^. Among the many intersections between these two systems, the link between cardiovascular disease (CVD) and Alzheimer’s disease (AD)—the most common form of dementia—is of particular interest^1,2^.

Both CVD and AD are age-associated chronic conditions that share overlapping risk factors including obesity, diabetes, smoking, environment, stress and sex differences^4,5^. Pathophysiologically, vascular damage from conditions like hypertension can disrupt the blood-brain barrier and lead to cerebral small vessel disease, manifesting as features such as white matter hyperintensities and microinfarcts^6^, which are associated with cognitive decline^7^. Conversely, forms of AD pathology like β-amyloid (Aβ) plaques and tau deposition may impair the regulation of autonomic nervous system and induce damage to cardiovascular function^4,8^. Despite this known interplay, the differential impact of specific CVD subtypes on AD risk remains poorly understood.

Large-scale population biobanks like the UK Biobank (UKB)^9^ and the All of Us Research Program (AoU)^10^ offer an unprecedented opportunity to clarify these relationships through robust, high-powered analyses across diverse populations^11^. Prior studies have examine individual CVD traits in relation to cognitive decline or AD, but comprehensive, subtype-level comparisons are limited^12–16^. This study aims to fill that gap by examining associations between AD and a wide range of CVD subtypes in both UKB and AoU, while also exploring shared genetic architecture through genome-wide association study (GWAS) data. Understanding which CVD subtypes are most strongly linked to AD may reveal key mechanistic pathways and inform targeted prevention strategies.

## Methods

### Study Population

We utilized data from two large, population-based biobanks: the UK Biobank (UKB) and the All of Us Research Program (AoU). UKB is a prospective cohort of 502,133 participants recruited from 22 centers across the UK between 2006 and 2010. The UKB resource is open to all bona fide researchers. Full details of its design and conduct are available online (https://www.ukbiobank.ac.uk). UKB received ethical approval from the National Health Service (NHS) Research Ethics Service (11/NW/0382); we conducted this analysis under application number 61915. All participants provided written informed consent, and the research was conducted in line with the Declaration of Helsinki.

AoU is a U.S.-based cohort established in 2015, with over 746,000 enrolled participants, of whom 287,011 had linked electronic health record (EHR) and survey data necessary for this study. All of Us Research Program is reviewed by an internal human subjects review board and all participants provide informed consent. Secondary analyses of de-identified data from AoU is not considered human subjects research.

### Phenotype Ascertainment

AD and CVD subtypes were identified via International Classification of Diseases, 10th Revision (ICD-10)^17^ codes from EHRs. We included the 11 CVD subtypes with ≥10,000 cases in UKB: hypertension (I10), hypotension (I95), angina pectoris (I20), acute myocardial infarction (I21), pulmonary embolism (I26), atrial fibrillation (I48), heart failure (I50), atrioventricular/left bundle-branch blockages, which are subsequently referred to as “blockage” (I44), chronic rheumatic heart disease (I05–I09), chronic ischemic heart disease (I25), and cerebral infarction (I63). AD was defined using ICD-10 code G30.

### Statistical Analysis

We performed cross-sectional logistic regression to estimate the odds ratios (ORs) of AD associated with each CVD subtype. Models were adjusted for key covariates affecting heart and brain health: age at assessment, sex, smoking status, education (age completed full-time education), depression status, physical activity (moderate and vigorous days/week), alcohol consumption, ethnicity, body mass index (BMI), annual income, and type 2 diabetes status^1,4,18,19^.

In UKB, most covariates were self-reported via baseline touchscreen questionnaire at the first visit to the assessment center^20^. BMI was calculated from height and weight measurements at first visit, diabetes status was determined through clinical records (E11), and depression was classified as “Yes” for a positive response to any type of depression or bipolar disorder, and “No” otherwise. All ethnic subgroups within the “White”, “Black”, “Mixed”, and “Asian” categories were described as their broader classification. All other variables retained their original levels from the UK BioBank^21^. Missing continuous covariate values were imputed using the median^22^ and categorical NAs were grouped as “Prefer not to answer.”

The same models were applied using AoU data where possible. We were not able to calculate the OR for pulmonary embolism, as there were only two positive cases with AD, making estimates highly unstable. Due to limited data availability, physical activity was excluded. BMI was averaged from all values reported for each participant. Age was estimated as years since birth as of 2025. Depression and diabetes were identified using ICD-10 codes. Ethnicity, smoking, income, alcohol use, and sex at birth were derived from self-reports, with “Prefer not to answer” and “Skip” responses combined. All individuals who listed multiple ethnicities were classified as “Mixed”.

### Genetic Analysis

To explore shared genetic architecture, we examined proximity between significant single nucleotide polymorphisms (SNPs) from UKB CVD GWASs performed by Backman et al.^23^ and SNPs from published GWASs related to brain structure and function included in the National Human Genome Research Institute-European Bioinformatics Institute (NHGRI-EBI) Catalog^24^ under the topics of dementia and all child traits, including AD, psychological disorder traits, and traits related to amyloid and tau levels. We assessed the reverse relationship too, as AD GWAS results from UKB were compared to catalog SNPs and cardiovascular disease traits, traits related to diastolic and systolic blood pressure, electrocardiogram derived traits, heart function, cardiac MRI values, heart rate, and heart shape measurements.

We also compared AD GWAS results from the catalog to GWAS results for 82 cardiac magnetic resonance imaging (CMR) traits in UKB. These CMR traits were derived through previous research and were return to UKB in the category “Cardiac and aortic function #1”. The cohort used for GWAS analysis for these traits was filtered to exclude participants with varying genetic and reported sex, non-white British ancestry, sex chromosome aneuploidy, and relatives. After filtering, 26,335 participants remained in the discovery dataset. We adjusted for the same covariates considered in previous research by Zhao et al^25^.

All GWASs used GRCh38 genome build^26^. We filtered for SNPs with p < 5×10^−8^ and identified colocalizations as SNP pairs located within 50 kb on the same chromosome. We then utilized NHGRI-EBI to search for genes containing these SNPs.

## Results

### Demographic Characteristics

In both the UK Biobank (UKB) and All of Us (AoU) cohorts, participants with Alzheimer’s disease (AD) were generally older, more likely to have diabetes, and had lower educational attainment and income compared to those without AD. AD cases were also more likely to be former or current smokers and less likely to report frequent alcohol consumption. BMI differences were minimal between groups. Ethnic representation was broader in AoU, while UKB was predominantly White (94.1%). These findings are summarized in Table 1 and Table 2.

### Cardiovascular Disease Subtypes and AD Prevalence

CVD subtypes showed varying prevalence across cohorts. In UKB, hypertension was most common (32.3%), followed by chronic ischemic heart disease and atrial fibrillation. In AoU, the distribution was similar, with slightly higher prevalence of hypertension (36.6%). AD prevalence was higher among individuals with each CVD subtype compared to those without, with the strongest differences seen in hypotension, heart failure, and cerebral infarction (Table 3 and Table 4).

### Associations Between CVD Subtypes and AD

**Figure 1 -:**
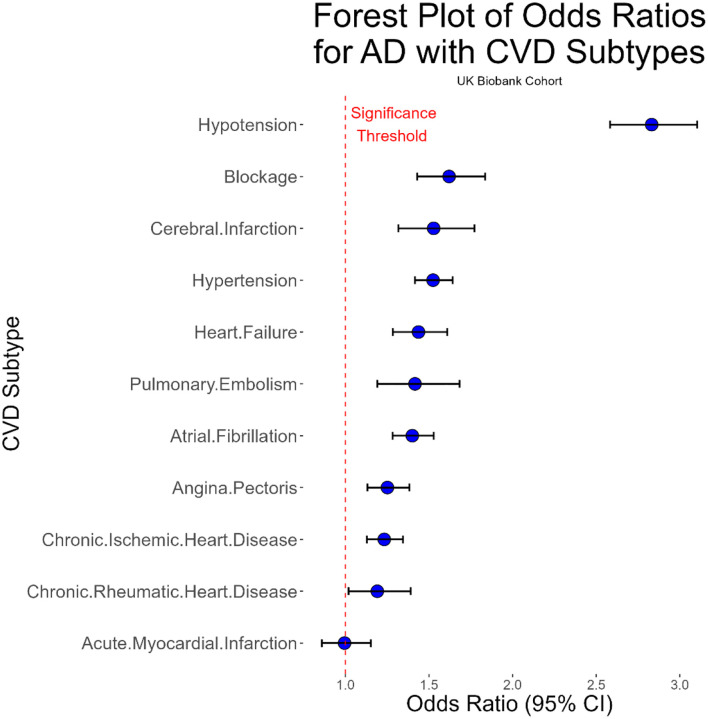
Forest plot of odds ratios for AD with CVD subtypes in the UKB cohort. Blue dots represent odds ratio point estimates, and error bars show the 95% confidence interval. The dotted red line shows the significance threshold. By far the strongest association was noted between hypotension and AD. All CVD subtypes besides acute myocardial infarction had significant relationships with AD.

**Figure 2 –:**
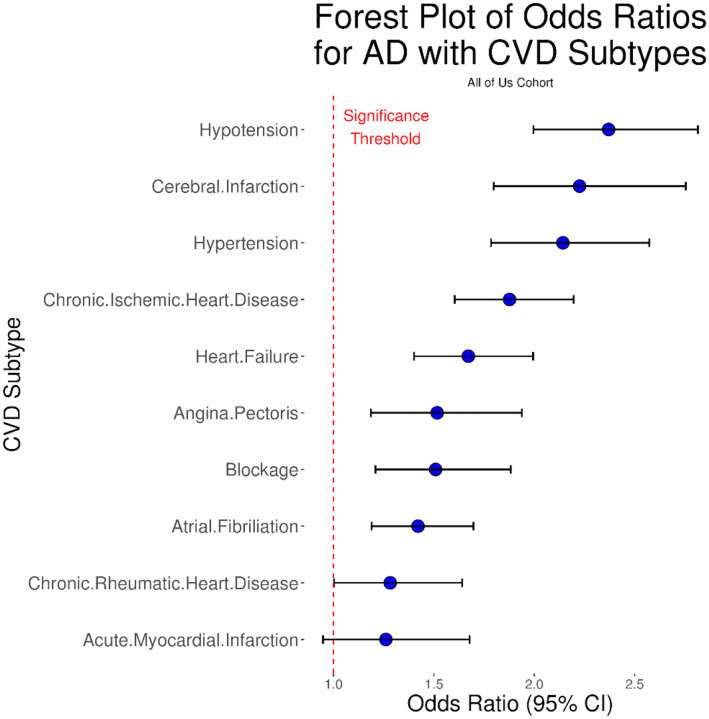
Forest plot of odds ratios for AD with CVD subtypes in the All of Us cohort. Blue dots represent odds ratio point estimates, and error bars show the 95% confidence interval. The dotted red line shows the significance threshold. Note the strongest associations between AD and hypotention, cerebral infarction, and hypertension. All CVD subtypes were significant at the 95% confidence level except for acute myocardial infarction.

Logistic regression analysis adjusted for relevant covariates revealed that nearly all CVD subtypes were significantly associated with higher odds of AD in both cohorts (Figure 1 and Figure 2). In UKB, the strongest association was observed between hypotension and AD (OR = 2.74, 95% CI: [2.52, 2.98]), followed by blockages (OR = 1.62, 95% CI: [1.45, 1.82]), hypertension (OR = 1.57, 95% CI: [1.47, 1.68]), and cerebral infarction (OR = 1.49, 95% CI: [1.30, 1.71]). Acute myocardial infarction was the only subtype not significantly associated with AD (OR = 1.01, 95% CI: [0.89, 1.15]).

Findings in AoU mirrored those from UKB. Again, hypotension showed the strongest association with AD (OR = 2.37, 95% CI: [2.00, 2.82], followed by cerebral infarction (OR = 2.23, 95% CI: [1.80, 2.75]) and hypertension (OR = 2.14, 95% CI: [1.78, 2.57]). AMI remained non-significant (OR = 1.26, 95% CI: [0.95, 1.68]). Odds ratio estimates in AoU had wider confidence intervals, likely reflecting the greater racial/ethnic diversity, broader socioeconomic representation, and the lack of adjustment for physical activity.

### Genetic Overlap Between CVD and AD

To explore shared genetic architecture, we identified SNPs significantly associated with both CVD and AD-related traits that were located within 50 kb of one another (p < 5×10^−8^). A total of 164 unique SNP pairs were found to be proximal across datasets, with the strongest overlaps observed between AD and traits such as angina pectoris, left ventricular myocardial wall thickness, and coronary artery disease.

Several loci stood out for high overlap, including:

- **19q13.32**, encompassing *APOE*, *TOMM40*, *APOC1*, *APOC1P1*, *NECTIN2*, and *APOC4-APOC2*, linked to lipid metabolism and both AD and cardiovascular traits^27–30^.
- **17q21.31**, home to *MAPT*, *KANSL1*, and *WNT3* with links to ventricular wall thickness and AD risk^25^.
- **11p11.2**, containing *PSMC3*, *SPI1*, and *RAPSN*, genes implicated in neuroinflammation, immune response, and cardiac structure^31,32^.

## Discussion

This study used two large, demographically distinct biobank datasets—UKB and AoU—to investigate associations between AD and multiple CVD subtypes. Our findings demonstrate that most CVD subtypes are significantly associated with higher odds of AD, with hypotension emerging as the strongest and most consistent correlation in both cohorts. These results highlight the importance of examining the heart–brain axis beyond general CVD risk and underscore the value of differentiating CVD subtypes in dementia research.

Notably, hypotension showed the highest odds of AD across both datasets. While hypertension has been extensively studied as a modifiable AD risk factor, hypotension is comparatively understudied despite its high prevalence among older adults^33^. Both chronic and orthostatic hypotension have been associated with cerebral hypoperfusion, oxidative stress, and tau pathology—mechanisms that could exacerbate or accelerate AD progression^2,13^. Conversely, AD-related dysfunction of the autonomic nervous system may impair cardiovascular regulation, suggesting a bidirectional relationship^34^.

Hypertension and cerebral infarction also demonstrated strong associations with AD, consistent with established literature linking these conditions to vascular pathology^2,4,15,35,36^, white matter damage^7^, and cognitive decline^5,37^. Atrial fibrillation, known to increase stroke risk and independently linked to cognitive impairment, was similarly associated with AD^16,38^. These findings support a broader vascular contribution to AD and indicate that multiple CVD pathways, particularly those impacting cerebral blood flow, may converge in AD pathophysiology.

The only CVD subtype not significantly associated with AD was acute myocardial infarction (AMI). This aligns with prior research suggesting that while AMI may contribute to cognitive decline through systemic inflammation or hypoxia^39^, its direct link to AD pathology remains limited^14,40^. However, AMI’s indirect effects on brain health warrant further investigation, particularly regarding rates of long-term cognitive decline post-infarction^39,41,42^.

Our genetic colocalization analysis identified overlapping SNPs between CVD and AD traits, especially in loci involving *APOE*, *MAPT*, *SPI1*, and *WNT3*. The *APOE* region (19q13.32) remains the most prominent shared locus, given its well-established roles in lipid metabolism^27,29^, blood-brain barrier integrity^6,28^, and both cardiovascular and neurodegenerative diseases^27,28,30^. Interestingly, we also observed overlap between myocardial wall thickness traits and AD-related SNPs in several regions, including 17q21.31 (*MAPT*) and 11p11.2 (*PSMC3*, *RAPSN*), pointing to potential shared mechanisms involving cardiac remodeling and brain structure integrity^25,43,44^.

Differences between the two cohorts—UKB’s healthier, less diverse sample versus AoU’s more representative and clinically diverse population—underscore the generalizability of our findings. The replicated associations across these distinct cohorts strengthen the robustness of our results and highlight the need for tailored prevention strategies that address specific CVD subtypes in diverse populations.

Despite the strengths of large-scale, multi-cohort replication and integration of genetic data, this study has limitations. First, its cross-sectional design precludes causal inference, and the directionality of associations cannot be determined. Second, AD and CVD diagnoses were based on ICD-10 codes, which may underrepresent true disease prevalence due to undiagnosed or misclassified cases. Third, we did not adjust for cardiovascular multimorbidity, and participants with multiple CVD subtypes may have higher cumulative AD risk^45,46^. Additionally, our genetic analysis, while informative, used proximity-based colocalization rather than formal methods such as Mendelian randomization or transcriptome-wide association studies, which could better infer causality or gene expression effects.

## Conclusion

In this study, we investigated the relationship between AD and multiple CVD subtypes across two large, diverse biobank cohorts: the UK Biobank and All of Us. We found that most CVD subtypes were significantly associated with AD, with hypotension showing the strongest association in both datasets. Other conditions like hypertension, cerebral infarction, and atrial fibrillation also demonstrated consistent positive associations, reinforcing the central role of vascular health in cognitive decline. Acute myocardial infarction was the only subtype not significantly linked to AD, consistent with prior literature.

Genetic analyses revealed that several AD- and CVD-associated SNPs were located near each other, especially in regions containing *APOE*, *MAPT*, and genes involved in myocardial structure. These results suggest a potential shared genetic basis between heart and brain pathology. While the cross-sectional design and reliance on ICD-10 codes limit causal inference, our findings underscore the importance of cardiovascular health in AD risk and highlight specific CVD subtypes that may warrant increased attention in prevention strategies.

## Figures and Tables

**Table 1 –:** Demographic information about UKB full cohort and split by AD status

Covariate	Status	Full Cohort	Without AD	With AD
Diabetes	No	457702 (91.2%)	454399 (91.2%)	3303 (80.0%)
	Yes	44431 (8.8%)	43605 (8.8%)	826 (20.0%)
Age		56.53 (8.09)	56.46 (8.08)	64.65 (4.24)
Sex	Female	273158 (54.4%)	271004 (54.4%)	2154 (52.2%)
	Male	228975 (45.6%)	227000 (45.6%)	1975 (47.8%)
Age completed full time education		16.36 (2.83)	16.36 (3.45)	15.71 (3.54)
	NA	165587 (33.0%)	164722 (33.1%)	865 (20.9%)
BMI		27.43 (4.80)	27.43 (4.80)	27.47 (4.69)
	NA	3103 (0.6%)	3063 (0.6%)	40 (1.0%)
Smoking Status	Never	273328 (54.4%)	271336 (54.5%)	1992 (48.2%)
	Previous	172920 (34.4%)	171206 (34.4%)	1714 (41.5%)
	Current	52937 (10.5%)	52566 (10.6%)	371 (9.0%)
	Prefer not to answer	2948 (0.6%)	2896 (0.6%)	52 (1.3%)
Depression/Bipolar Disorder	No	468716 (93.3%)	464772 (93.3%)	3944 (95.5%)
	Yes	33417 (6.7%)	33232 (6.7%)	185 (4.5%)
Days of moderate physical activity each week		3.63 (2.33)	3.62 (2.33)	3.95 (2.38)
	NA	27251 (5.4%)	26876 (5.4%)	375 (9.1%)
Days of vigorous physical activity each week		1.84 (1.96)	1.84 (1.96)	1.85 (2.12)
	NA	27565 (5.5%)	27157 (5.5%)	408 (9.9%)
Alcohol Consumption	Daily or almost daily	603 (0.1%)	595 (0.1%)	8 (0.2%)
	3–4 times a week	101716 (20.3%)	100933 (20.3%)	783 (19.0%)
	1–2 times a week	115357 (23.0%)	114552 (23.0%)	805 (19.5%)
	1–3 times a month	129186 (25.7%)	128215 (25.7%)	971 (23.5%)
	Special occasions only	55811 (11.1%)	55392 (11.1%)	419 (10.1%)
	Never	57967 (11.5%)	57379 (11.5%)	588 (14.2%)
	Prefer not to answer	41493 (8.3%)	40938 (8.2%)	555 (13.4%)
Annual Household Income	<£18,000	49778 (9.9%)	49203 (9.9%)	575 (13.9%)
	£18,000–£30,999	21295 (4.2%)	20900 (4.2%)	395 (9.6%)
	£31,000–£51,999	97143 (19.3%)	95768 (19.2%)	1375 (33.3%)
	£52,000–£100,000	108101 (21.5%)	107135 (21.5%)	966 (23.4%)
	>£100,000	110690 (22.0%)	110226 (22.1%)	464 (11.2%)
	Do not know	86204 (17.2%)	86009 (17.3%)	195 (4.7%)
	Prefer not to answer	28922 (5.8%)	28763 (5.8%)	159 (3.9%)
Ethnic background	White	472365 (94.1%)	468415 (94.1%)	3950 (95.7%)
	Black	8048 (1.6%)	7990 (1.6%)	58 (1.4%)
	Asian	9872 (2.0%)	9818 (2.0%)	54 (1.3%)
	Chinese	1571 (0.3%)	1567 (0.3%)	4 (0.1%)
	Mixed	2950 (0.6%)	2937 (0.6%)	13 (0.3%)
	Other	4552 (0.9%)	4529 (0.9%)	23 (0.6%)
	Unknown	217 (0.0%)	216 (0.0%)	1 (0.0%)
	Prefer not to answer	2558 (0.5%)	2532 (0.5%)	26 (0.6%)

**Table 2 -:** Demographic information about All of Us full cohort and split by AD status

Covariate	Status	Full Cohort	Without AD	With AD
Diabetes	No	237281 (82.7%)	236807 (82.7%)	474 (61.2%)
	Yes	49730 (17.3%)	49430 (17.3%)	300 (38.8%)
Age		58.01 (16.96)	57.95 (16.93)	79.95 (9.88)
Sex	Female	172401 (60.1%)	171999 (60.1%)	402 (51.9%)
	Male	108739 (37.9%)	108388 (37.9%)	351 (45.3%)
	Intersex	57 (0.0%)	57 (0.0%)	0 (0.0%)
	No matching concept	2702 (0.9%)	2692 (0.9%)	10 (1.3%)
	None	96 (0.0%)	96 (0.0%)	0 (0.0%)
	Prefer not to answer	3016 (1.1%)	3005 (1.0%)	11 (1.4%)
Highest Level of Education	Never Attended	421 (0.1%)	416 (0.1%)	5 (0.6%)
	Grades 1–4	2548 (0.9%)	2532 (0.9%)	16 (2.1%)
	Grades 5–8	6745 (2.4%)	6700 (2.3%)	45 (5.8%)
	Grades 9–11	18153 (6.3%)	18115 (6.3%)	38 (4.9%)
	Grade 12 Or GED	56778 (19.8%)	56647 (19.8%)	131 (16.9%)
	1–3 Years College	72938 (25.4%)	72780 (25.4%)	158 (20.4%)
	College Graduate	62550 (21.8%)	62394 (21.8%)	156 (20.2%)
	Advanced Degree	57696 (20.1%)	57494 (20.1%)	202 (26.1%)
	Prefer not to answer	9182 (3.2%)	9159 (3.2%)	23 (3.0%)
BMI		29.90 (7.64)	29.90 (7.64)	28.65 (6.57)
	NA	12715 (4.4%)	12676 (4.4%)	39 (5.0%)
Smoked 100 Cigarettes in Lifetime	No	164021 (57.1%)	163612 (57.2%)	409 (52.8%)
	Yes	114595 (39.9%)	114253 (39.9%)	342 (44.2%)
	Don’t Know	3645 (1.3%)	3636 (1.3%)	9 (1.2%)
	Prefer not to answer	4750 (1.7%)	4736 (1.7%)	14 (1.8%)
Depression	No	208532 (72.7%)	208193 (72.7%)	339 (43.8%)
	Yes	78479 (27.3%)	78044 (27.3%)	435 (56.2%)
Frequency of Alcoholic Drinks over Past Year	4 or More Per Week	30087 (10.5%)	30003 (10.5%)	84 (10.9%)
	2 to 3 Per Week	34981 (12.2%)	34920 (12.2%)	61 (7.9%)
	2 to 4 Per Month	52212 (18.2%)	52117 (18.2%)	95 (12.3%)
	Monthly Or Less	84407 (29.4%)	84226 (29.4%)	181 (23.4%)
	Never	46653 (16.3%)	46422 (16.2%)	231 (29.8%)
	Prefer not to answer	38671 (13.5%)	38549 (13.5%)	122 (15.8%)
Annual Household Income	<$10,000	40856 (14.2%)	40781 (14.2%)	75 (9.7%)
	$10,000–$25,000	33965 (11.8%)	33851 (11.8%)	114 (14.7%)
	$25,000–$35,000	20267 (7.1%)	20216 (7.1%)	51 (6.6%)
	$35,000–$50,000	22252 (7.8%)	22184 (7.8%)	68 (8.8%)
	$50,000–$75,000	29186 (10.2%)	29106 (10.2%)	80 (10.3%)
	$75,000–$100,000	22359 (7.8%)	22308 (7.8%)	51 (6.6%)
	$100,000–$150,000	27202 (9.5%)	27146 (9.5%)	56 (7.2%)
	$150,000–$200,000	12496 (4.4%)	12472 (4.4%)	24 (3.1%)
	>$200,000	17507 (6.1%)	17480 (6.1%)	27 (3.5%)
	Prefer not to answer	60921 (21.2%)	60693 (21.2%)	228 (29.5%)
Ethnicity	White	150402 (52.4%)	149897 (52.4%)	505 (65.2%)
	Black	57177 (19.9%)	57088 (19.9%)	89 (11.5%)
	Hispanic	47699 (16.6%)	47579 (16.6%)	120 (15.5%)
	Asian	8038 (2.8%)	8025 (2.8%)	13 (1.7%)
	MENA	1605 (0.6%)	1603 (0.6%)	2 (0.3%)
	NHPI	297 (0.1%)	296 (0.1%)	1 (0.1%)
	Mixed	10771 (3.8%)	10755 (3.8%)	16 (2.1%)
	None Of These	3017 (1.1%)	3017 (1.1%)	0 (0.0%)
	Prefer not to answer	8005 (2.8%)	7977 (2.8%)	28 (3.6%)

**Table 3 –:** Counts of UKB participants with each subtype of CVD overall and split by AD status

CVD Subtype	CVD Status	n (%)	Without AD	With AD
Hypertension	No	339872 (67.7%)	338205 (99.5%)	1667 (0.5%)
	Yes	162261 (32.3%)	159799 (98.5%)	2462 (1.5%)
Hypotension	No	480823 (95.8%)	477440 (99.3%)	3383 (0.7%)
	Yes	21310 (4.2%)	20564 (96.5%)	746 (3.5%)
Angina Pectoris	No	467966 (93.2%)	464473 (99.3%)	3493 (0.7%)
	Yes	34167 (6.8%)	33531 (98.1%)	636 (1.9%)
Acute Myocardial Infarction	No	484311 (96.5%)	480433 (99.2%)	3878 (0.8%)
	Yes	17822 (3.5%)	17571 (98.6%)	251 (1.4%)
Pulmonary Embolism	No	492066 (98.0%)	488111 (99.2%)	3955 (0.8%)
	Yes	10067 (2.0%)	9893 (98.3%)	174 (1.7%)
Atrial Fibrillation	No	460772 (91.8%)	457474 (99.3%)	3298 (0.7%)
	Yes	41361 (8.2%)	40530 (98%)	831 (2%)
Heart Failure	No	482058 (96.0%)	478397 (99.2%)	3661 (0.8%)
	Yes	20075 (4.0%)	19607 (97.7%)	468 (2.3%)
Blockage	No	488114 (97.2%)	484345 (99.2%)	3769 (0.8%)
	Yes	14019 (2.8%)	13659 (97.4%)	360 (2.6%)
Chronic Rheumatic Heart Disease	No	490849 (97.8%)	486930 (99.2%)	3919 (0.8%)
	Yes	11284 (2.2%)	11074 (98.1%)	210 (1.9%)
Chronic Ischemic Heart Disease	No	448703 (89.4%)	445508 (99.3%)	3195 (0.7%)
	Yes	53430 (10.6%)	52496 (98.3%)	934 (1.7%)
Cerebral Infarction	No	491788 (97.9%)	487900 (99.2%)	3888 (0.8%)
	Yes	10345 (2.1%)	10104 (97.7%)	241 (2.3%)

**Table 4 -:** Counts of AoU participants with each subtype of CVD overall and split by AD status

CVD Subtype	CVD Status	n (%)	Without AD	With AD
Hypertension	No	182015 (63.4%)	181840 (99.9%)	175 (0.1%)
	Yes	104996 (36.6%)	104397 (99.4%)	599 (0.6%)
Hypotension	No	269538 (93.9%)	268956 (99.8%)	582 (0.2%)
	Yes	17473 (6.1%)	17281 (98.9%)	192 (1.1%)
Angina Pectoris	No	278021 (96.9%)	277322 (99.7%)	699 (0.3%)
	Yes	8990 (3.1%)	8915 (99.2%)	75 (0.8%)
Acute Myocardial Infarction	No	280431 (97.7%)	279711 (99.7%)	720 (0.3%)
	Yes	6580 (2.3%)	6526 (99.2%)	54 (0.8%)
Atrial Fibrillation	No	270197 (94.1%)	269607 (99.8%)	590 (0.2%)
	Yes	16814 (5.9%)	16630 (98.9%)	184 (1.1%)
Heart Failure	No	268407 (93.5%)	267828 (99.8%)	579 (0.2%)
	Yes	18604 (6.5%)	18409 (99%)	195 (1%)
Blockage	No	278876 (97.2%)	278201 (99.8%)	675 (0.2%)
	Yes	8135 (2.8%)	8036 (98.8%)	99 (1.2%)
Chronic Rheumatic Heart Disease	No	278976 (97.2%)	278277 (99.7%)	699 (0.3%)
	Yes	8035 (2.8%)	7960 (99.1%)	75 (0.9%)
Chronic Ischemic Heart Disease	No	254852 (88.8%)	254401 (99.8%)	451 (0.2%)
	Yes	32159 (11.2%)	31836 (99%)	323 (1%)
Cerebral Infarction	No	279084 (97.2%)	278417 (99.8%)	667 (0.2%)
	Yes	7927 (2.8%)	7820 (98.7%)	107 (1.3%)

**Table 5 -:** Proximal SNPs related to the heart and brain. Traits are reported associated with SNP in the catalog, as well as traits that had significant UKB GWAS results, the number of pairings with each catalog SNP, and the gene(s) associated with each SNP. Only SNPs with greater than 2 pairings are reported

Catalog SNP	Catalog Traits	UKB Traits	Number of Pairings	Associated Genes
rs143364530	Alzheimer’s disease or educational attainment (pleiotropy)	LV mean myocardial wall thickness AHA 11, LV mean myocardial wall thickness AHA 12, LV mean myocardial wall thickness global	42	*KANSL1*
rs12292911	Alzheimer’s disease or family history of Alzheimer’s disease	LV mean myocardial wall thickness AHA 5, LV mean myocardial wall thickness AHA 8, LV mean myocardial wall thickness global	23	*PSMC3, RAPSN*
rs4434960	Alzheimer’s disease	LV mean myocardial wall thickness AHA 5, LV mean myocardial wall thickness AHA 8, LV mean myocardial wall thickness global	23	*PSMC3, RAPSN*
rs10437655	Alzheimer’s disease (MTAG)	LV mean myocardial wall thickness AHA 5, LV mean myocardial wall thickness AHA 7, LV mean myocardial wall thickness AHA 8, LV mean myocardial wall thickness global	15	*SPI1*
rs73069394	Alzheimer’s disease (MTAG)	Ascending aorta maximum area, Ascending aorta minimum area	10	*ULK4*
rs1065712	Alzheimer’s disease	LV radial strain AHA 15	8	*CTSB*
rs3740688	Late-onset Alzheimer’s disease	LV mean myocardial wall thickness AHA 5, LV mean myocardial wall thickness AHA 7, LV mean myocardial wall thickness AHA 8, LV mean myocardial wall thickness global	8	*SPI1*
rs4420638	total cholesterol measurement, hematocrit, stroke, ventricular rate measurement, body mass index, atrial fibrillation, high density lipoprotein cholesterol measurement, coronary artery disease, diastolic blood pressure, triglyceride measurement, systolic blood pressure, heart failure, diabetes mellitus, glucose measurement, mortality, cancer, total cholesterol measurement, diastolic blood pressure, triglyceride measurement, systolic blood pressure, hematocrit, ventricular rate measurement, glucose measurement, body mass index, high density lipoprotein cholesterol measurement, Alzheimer disease, amyloid-beta measurement, Alzheimer’s disease biomarker measurement, Alzheimer’s disease biomarker measurement, t-tau measurement	Alzheimer’s disease, angina pectoris	7	*APOC1, APOC1P1*
rs2696697	Alzheimer’s disease polygenic risk score (upper quantile vs lower quantile)	LV mean myocardial wall thickness AHA 11, LV mean myocardial wall thickness AHA 12, LV mean myocardial wall thickness global	6	*KANSL1*
rs769449	coronary artery disease, diastolic blood pressure, Alzheimer disease, amyloid-beta measurement, t-tau measurement	Alzheimer’s disease, angina pectoris	6	*APOE*
rs1065853	occlusion precerebral artery, systolic blood pressure, Alzheimer disease, polygenic risk score	Alzheimer’s disease, angina pectoris	5	*APOE, APOC1*
rs429358	brain infarction, neuritic plaque measurement, Lewy body dementia, cerebral amyloid angiopathy, neurofibrillary tangles measurement, systolic blood pressure, Alzheimer disease, Lewy body dementia, atrophic macular degeneration, age-related macular degeneration, wet macular degeneration, t-tau measurement	Alzheimer’s disease, angina pectoris	5	*APOE*
rs157582	level of protein Wnt-10b in blood serum, Alzheimer disease, amyloid-beta measurement	Alzheimer’s disease, angina pectoris	4	*TOMM40*
rs2075650	body fat percentage, coronary artery disease, posterior cortical atrophy, Alzheimer disease, t-tau measurement	Alzheimer’s disease, angina pectoris	4	*TOMM40*
rs56131196	coronary artery disease, Alzheimer disease, Alzheimer disease, amyloid-beta measurement	Alzheimer’s disease, angina pectoris	4	*APOC1, APOC1P1*
rs113568679	Alzheimer’s disease or educational attainment (pleiotropy)	LV mean myocardial wall thickness AHA 11, LV mean myocardial wall thickness AHA 12, LV mean myocardial wall thickness global	3	*MAPT*
rs141622900	coronary artery disease, Alzheimer disease, high density lipoprotein cholesterol measurement	Alzheimer’s disease, angina pectoris	3	*APOC1, APOC1P1*
rs199498	Alzheimer’s disease	LV mean myocardial wall thickness AHA 11, LV mean myocardial wall thickness AHA 12, LV mean myocardial wall thickness global	3	*WNT3*
rs199503	Alzheimer’s disease or educational attainment (pleiotropy)	LV mean myocardial wall thickness AHA 11, LV mean myocardial wall thickness AHA 12, LV mean myocardial wall thickness global	3	*WNT3*
rs199515	Alzheimer’s disease	LV mean myocardial wall thickness AHA 11, LV mean myocardial wall thickness AHA 12, LV mean myocardial wall thickness global	3	*WNT3*
rs390082	level of protein S100-A13 in blood serum, Alzheimer disease	Alzheimer’s disease, angina pectoris	3	*APOE, APOC1*
rs41290120	myocardial infarction, Alzheimer disease, high density lipoprotein cholesterol measurement	Alzheimer’s disease, angina pectoris	3	*NECTIN2*
rs483082	Alzheimer disease, amyloid-beta measurement	angina pectoris	3	*APOE, APOC1*
rs5117	lobar intracerebral hemorrhage, cerebral amyloid angiopathy	Alzheimer’s disease, angina pectoris	3	*APOC1*
rs5167	level of hepatoma-derived growth factor-related protein 3 in blood serum, Alzheimer disease	Alzheimer’s disease, angina pectoris	3	*APOC4-APOC2, APOC4*
rs59007384	Alzheimer disease, amyloid-beta measurement	angina pectoris	3	*TOMM40*
rs6859	cardiovascular disease, Alzheimer disease	Alzheimer’s disease, angina pectoris	3	*NECTIN2*
rs7412	response to darapladib, lipoprotein-associated phospholipase A(2) change measurement,, systolic blood pressure	Alzheimer’s disease	3	*APOE*
rs814573	myocardial infarction, Alzheimer disease, polygenic risk score	Alzheimer’s disease, angina pectoris	3	*APOC1, APOC1P1*

## Data Availability

The computer source code for this study is available at https://github.com/MIILab-MTU/CVDSubtypesADCorrelation.
